# Population clustering of structural brain aging and its association with brain development

**DOI:** 10.7554/eLife.94970

**Published:** 2024-10-18

**Authors:** Haojing Duan, Runye Shi, Jujiao Kang, Tobias Banaschewski, Arun LW Bokde, Christian Büchel, Sylvane Desrivières, Herta Flor, Antoine Grigis, Hugh Garavan, Penny A Gowland, Andreas Heinz, Rüdiger Brühl, Jean-Luc Martinot, Marie-Laure Paillère Martinot, Eric Artiges, Frauke Nees, Dimitri Papadopoulos Orfanos, Luise Poustka, Sarah Hohmann, Nathalie Nathalie Holz, Juliane Fröhner, Michael N Smolka, Nilakshi Vaidya, Henrik Walter, Robert Whelan, Gunter Schumann, Xiaolei Lin, Jianfeng Feng

**Affiliations:** 1 Institute of Science and Technology for Brain-Inspired Intelligence, Fudan University Shanghai China; 2 Key Laboratory of Computational Neuroscience and Brain-Inspired Intelligence (Fudan University), Ministry of Education Shanghai China; 3 School of Data Science, Fudan University Shanghai China; 4 Department of Child and Adolescent Psychiatry and Psychotherapy, Central Institute of Mental Health, Medical Faculty Mannheim, Heidelberg University Mannheim Germany; 5 Discipline of Psychiatry, School of Medicine and Trinity College Institute of Neuroscience, Trinity College Dublin Dublin Ireland; 6 University Medical Centre Hamburg-Eppendorf Hamburg Germany; 7 Social Genetic and Developmental Psychiatry Centre, Institute of Psychiatry, Psychology and Neuroscience, King’s College London London United Kingdom; 8 Institute of Cognitive and Clinical Neuroscience, Central Institute of Mental Health, Medical Faculty Mannheim, Heidelberg University Mannheim Germany; 9 Department of Psychology, School of Social Sciences, University of Mannheim Mannheim Germany; 10 NeuroSpin, CEA, Université Paris-Saclay Gif-sur-Yvette France; 11 Departments of Psychiatry and Psychology, University of Vermont Burlington United States; 12 Sir Peter Mansfield Imaging Centre School of Physics and Astronomy, University of Nottingham Nottingham United Kingdom; 13 Department of Psychiatry and Psychotherapy CCM, Charité – Universitätsmedizin Berlin, corporate member of Freie Universität Berlin, Humboldt-Universität zu Berlin, and Berlin Institute of Health Berlin Germany; 14 Physikalisch-Technische Bundesanstalt (PTB), Braunschweig and Berlin Berlin Germany; 15 Institut National de la Santé et de la Recherche Médicale, INSERM U1299 "Developmental Trajectories and Psychiatry", Université Paris-Saclay, Ecole Normale supérieure Paris-Saclay, CNRS, Centre Borelli Gif-sur-Yvette France; 16 AP-HP. Sorbonne Université, Department of Child and Adolescent Psychiatry, Pitié-Salpêtrière Hospital Paris France; 17 Psychiatry Department, EPS Barthélémy Durand Etampes France; 18 Institute of Medical Psychology and Medical Sociology, University Medical Center Schleswig-Holstein, Kiel University Kiel Germany; 19 Department of Child and Adolescent Psychiatry and Psychotherapy, University Medical Centre Göttingen Germany; 20 Department of Psychiatry and Neuroimaging Center, Technische Universität Dresden Dresden Germany; 21 Department of Psychiatry and Neurosciences, Charité–Universitätsmedizin Berlin, corporate member of Freie Universität BerlinHumboldt-Universität zu Berlin, and Berlin Institute of Health Berlin Germany; 22 School of Psychology and Global Brain Health Institute, Trinity College Dublin Dublin Ireland; 23 Centre for Population Neuroscience and Stratified Medicine (PONS Centre), ISTBI, Fudan University Shanghai China; 24 Centre for Population Neuroscience and Stratified Medicine (PONS), Department of Psychiatry and Psychotherapy, Charité Universitätsmedizin Berlin Germany; 25 Huashan Institute of Medicine, Huashan Hospital affiliated to Fudan University Shanghai China; 26 MOE Frontiers Center for Brain Science, Fudan University Shanghai China; 27 Zhangjiang Fudan International Innovation Center Shanghai China; 28 Department of Computer Science, University of Warwick Warwick United Kingdom; Seoul National University Republic of Korea; Seoul National University Republic of Korea

**Keywords:** structural brain aging, adolescence, longitudinal analysis, MRI, genetics, Human

## Abstract

Structural brain aging has demonstrated strong inter-individual heterogeneity and mirroring patterns with brain development. However, due to the lack of large-scale longitudinal neuroimaging studies, most of the existing research focused on the cross-sectional changes of brain aging. In this investigation, we present a data-driven approach that incorporate both cross-sectional changes and longitudinal trajectories of structural brain aging and identified two brain aging patterns among 37,013 healthy participants from UK Biobank. Participants with accelerated brain aging also demonstrated accelerated biological aging, cognitive decline and increased genetic susceptibilities to major neuropsychiatric disorders. Further, by integrating longitudinal neuroimaging studies from a multi-center adolescent cohort, we validated the ‘last in, first out’ mirroring hypothesis and identified brain regions with manifested mirroring patterns between brain aging and brain development. Genomic analyses revealed risk loci and genes contributing to accelerated brain aging and delayed brain development, providing molecular basis for elucidating the biological mechanisms underlying brain aging and related disorders.

## Introduction

The structure of the brain undergoes continual changes throughout the entire lifespan, with structural brain alterations intimately linking brain development and brain aging (Fjell and Walhovd, 2010; Shaw et al., 2008). Brain aging is a progressive process that often co-occurs with biological aging and declines of cognitive functions (Elliott et al., 2021; Mattson and Arumugam, 2018; Park and Reuter-Lorenz, 2009), which contribute to the onset and acceleration of neurodegenerative (Mariani et al., 2005) and neuropsychiatric disorders (Kaufmann et al., 2019). Studies on healthy brain aging have revealed significant inter-individual heterogeneity in the patterns of neuroanatomical changes (Raz et al., 2010; Raz and Rodrigue, 2006). Therefore, examining the patterns of structural brain aging and its associations with cognitive decline is of paramount importance in understanding the diverse biological mechanisms of age-related neuropsychiatric disorders.

Despite the fact that there exist large differences between brain development and brain aging (Courchesne et al., 2000), a discernible association between these two processes remains evident. Direct comparisons of brain development and brain aging using structural MRI indicated a ‘last in, first out’ mirroring pattern, where brain regions develop relatively late during adolescence demonstrated accelerated degeneration in older ages (McGinnis et al., 2011; Tamnes et al., 2013). In addition, brain regions with strong mirroring effects showed increased vulnerability to neurodegenerative and neuropsychiatric disorders, including Alzheimer’s disease and schizophrenia (Douaud et al., 2014). However, due to the lack of large-scale longitudinal MRI studies during adolescence and mid-to-late adulthood, validation of the ‘last in, first out’ mirroring hypothesis remains unavailable.

Prior investigations have largely focused on regional and cross-sectional changes of brain aging (Raz and Rodrigue, 2006; Douaud et al., 2014; Suzuki et al., 2019), with relatively few studies exploring longitudinal trajectories of brain aging and its associations with brain development (Raz et al., 2010; Fjell et al., 2015; Nyberg et al., 2023). In this article, we present a data-driven approach to examine the population clustering of longitudinal brain aging trajectories using structure MRI data obtained from 37,013 healthy individuals during mid-to-late adulthood (44–82 years), and explore its association with biological aging, cognitive decline and susceptibilities for neuropsychiatric disorders. Further, mirroring patterns between longitudinal brain development and brain aging are investigated by comparing the region-specific aging / developmental trajectories, and manifestation of the mirroring patterns are investigated across the whole-brain and among participants with different brain aging patterns. Genomic analyses are conducted to reveal risk loci and genes associated with accelerated brain aging and delayed brain development.

## Results

### Longitudinal trajectories of whole-brain gray matter volume in mid-to-late adulthood define two brain aging patterns

**Figure 1.**
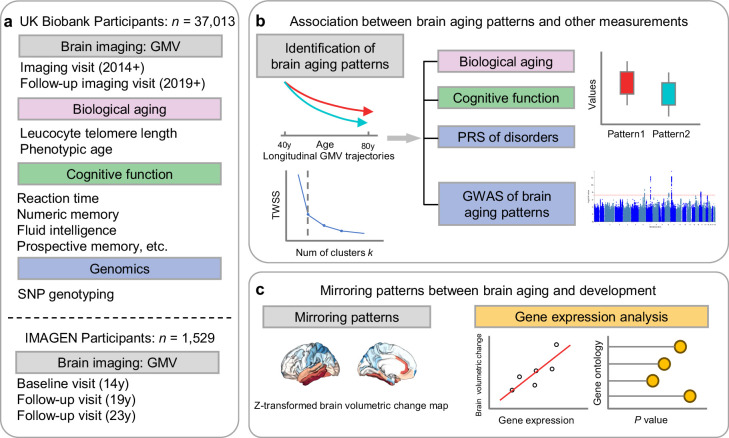
Overview of the study workflow. (**a**) Population cohorts (UK Biobank and IMAGEN) and data sources (brain imaging, biological aging biomarkers, cognitive functions, genomic data) involved in this study. (**b**) Brain aging patterns were identified using longitudinal trajectories of the whole brain GMV, which enabled the capturing of long-term and individualized variations compared to only use cross-sectional data, and associations between brain aging patterns and other measurements (biological aging, cognitive functions and PRS of major neuropsychiatric disorders) were investigated. (c) Mirroring patterns between brain aging and brain development was investigated using z-transformed brain volumetric change map and gene expression analysis.

**Figure 2.**
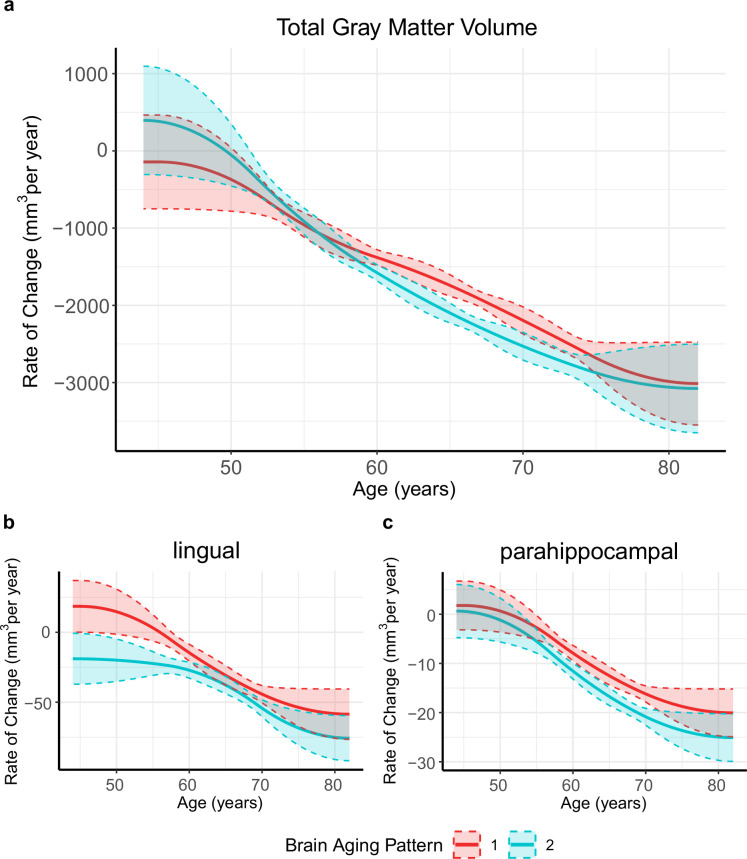
Global (**a**) and selected regional (**b, c**) cortical gray matter volume rate of change among participants with brain aging patterns 1 (red) and 2 (blue). Rates of volumetric change for total gray matter and each ROI were estimated using GAMM, which incorporates both cross-sectional between-subject variation and longitudinal within-subject variation from 40,921 observations and 37,013 participants. Covariates include sex, assessment center, handedness, ethnic, and ICV. Shaded areas around the fit line denotes 95% CI. Figure 2—source data 1.Related to Figure 2.Global (**a**) and selected regional (**b, c**) cortical gray matter volume rate of change among participants with brain aging patterns 1 (red) and 2 (blue).

Figure 1 provides the data sources, analytical workflow and research methodology of this study. After the sample selection process (Appendix 1—figure 1, Appendix 1—table 1 and 2), longitudinal grey matter volume (GMV) trajectories in 40 ROIs (33 cortical and 7 subcortical ROIs, see Appendix 1—table 3) were estimated for each of the 37,013 healthy participants in UK Biobank. The first 15 principal components derived from dimensionality reduction via principal component analysis were used in the clustering analysis (see Methods; Alexander-Bloch et al., 2013; Whitwell et al., 2009). Two brain aging patterns were identified, where 18,929 (51.1%) participants with the first brain aging pattern (pattern 1) had higher total GMV at baseline and a slower rate of GMV decrease over time, and the remaining participants with the second pattern (pattern 2) had lower total GMV at baseline and a faster rate of GMV decrease (Figure 2a). Comparing the region-specific rate of GMV decrease, pattern 2 showed a more rapid GMV decrease in medial occipital (lingual gyrus, cuneus, and pericalcarine cortex) and medial temporal (entorhinal cortex, parahippocampal gyrus) regions (Figure 2b and c and Appendix 1—figure 2), which had the largest loadings in the second and third principal components (Appendix 1—table 4). These two patterns can be clearly stratified by both linear and non-linear dimensionality reduction methods, indicating distinct structural differences in brain aging between patterns (Appendix 1—figure 3). Sample characteristics of these 37,013 UK Biobank participants stratified by brain aging patterns are summarized in Appendix 1—table 5. Overall, participants with different brain aging patterns had similar distributions with regard to age, sex, ethnicity, smoking status, Townsend deprivation index (TDI), body mass index (BMI) and years of schooling.

### Brain aging patterns were significantly associated with biological aging

**Figure 3.**
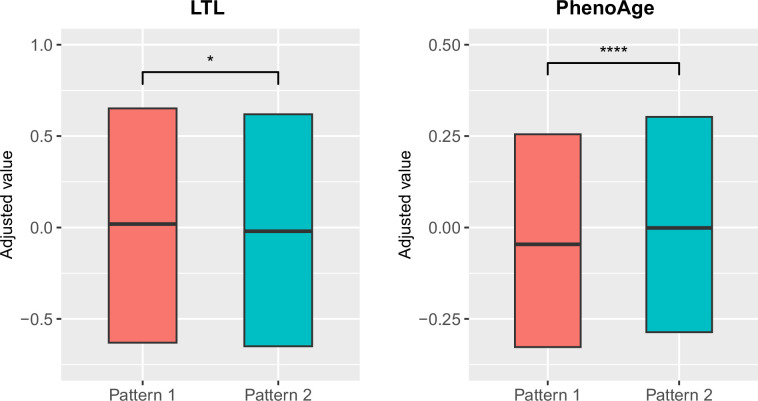
Distributions of biological aging biomarkers (leucocyte telomere length (LTL) and PhenoAge) among participants with brain aging patterns 1 and 2. Boxes represent the interquartile range (IQR), lines within the boxes indicate the median. Two-sided p values were obtained by comparing LTL or PhenoAge Levine et al., 2018 between brain aging patterns using unadjusted multivariate linear regression models. Results remained significant when adjusting for sex, age, ethnic, BMI, smoking status and alcohol intake frequency in the LTL model Demanelis et al., 2020 and sex, age, ethnic, BMI, smoking status, alcohol frequency and education years in PhenoAge model. Stars indicate statistical significance after Bonferroni correction. ****: p ≤ 0.0001, *: p ≤ 0.05. Figure 3—source data 1.Related to Figure 3.Distributions of biological aging biomarkers (leucocyte telomere length (LTL) and PhenoAge) among participants with brain aging patterns 1 and 2.

To explore the relationships between structural brain aging and biological aging, we investigated the distribution of aging biomarkers, such as telomere length and PhenoAge (Levine et al., 2018), across brain aging patterns identified above (Figure 3 and Appendix 1—table 6). Compared to pattern 1, participants in pattern 2 with more rapid GMV decrease had shorter leucocyte telomere length (p=0.009, Cohen’s D=–0.028) and this association remained consistent after adjusting for sex, age, ethnic, BMI, smoking status and alcohol intake frequency (Demanelis et al., 2020). Next, we examined PhenoAge, which was developed as an aging biomarker incorporating composite clinical and biochemical data (Levine et al., 2018), and observed higher PhenoAge among participants with brain aging pattern 2 compared to pattern 1 (p=0.019, Cohen’s D=0.027). Again, the association remained significant after adjusting for sex, age, ethnic, BMI, smoking status, alcohol intake frequency and education years (p=3.05×10^–15^, Cohen’s D=0.092). Group differences in terms of each individual component of PhenoAge (including albumin, creatinine, glucose, c-reactive protein, lymphocytes percentage, mean corpuscular volume, erythocyte distribution width, alkaline phosphatase and leukocyte count) were also investigated and results were consistent with PhenoAge (Appendix 1—figure 4).

### Accelerated brain aging was associated with cognitive decline and increased genetic susceptibilities to attention-deficit/hyperactivity disorder and delayed brain development

**Figure 4.**
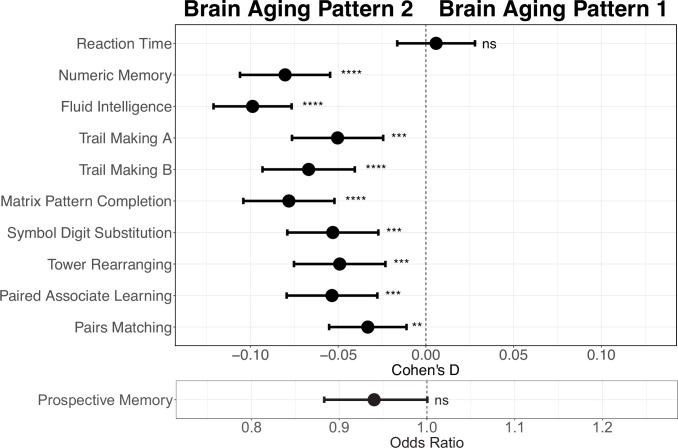
Effect size (Cohen’s D or odds ratio) for comparing the cognitive functions between participants with brain aging patterns 1 and 2. Results were adjusted such that negative Cohen’s D and Odds Ratio less than 1 indicate worse cognitive performances in brain aging pattern 2 compared to pattern 1. Width of the lines extending from the center point represent 95% confidence interval. Two-sided p values were obtained using both unadjusted and adjusted (for sex, age, and TDI, education and income) multivariate regression models. Stars indicate statistical significance after FDR correction for 11 comparisons. ****: p ≤ 0.0001, ***: p ≤ 0.001, **: p ≤ 0.01, ns: p>0.05. Figure 4—source data 1.Related to Figure 4.Effect size (Cohen’s D or odds ratio) for comparing the cognitive functions between participants with brain aging patterns 1 and 2.

Next, we conducted comprehensive comparisons of cognitive functions between participants with different brain aging patterns. In general, those with brain aging pattern 2 (lower baseline total GMV and more rapid GMV decrease) exhibited worse cognitive performances compared to pattern 1. Specifically, brain aging pattern 2 showed lower numbers of correct pairs matching (p=0.006, Cohen’s D=–0.029), worse prospective memory (OR = 0.943, 95% CI [0.891, 0.999]), lower fluid intelligence (p<1.00×10^–20^, Cohen’s D=–0.102), and worse numeric memory (p=5.97×10^–11^, Cohen’s D=–0.082). No statistically significant differences were observed in terms of the reaction time (p=0.99) and prospective memory (p=0.052) between these two brain aging patterns after FDR correction. Results were consistent when using models adjusted for sex, age, and socioeconomic status (TDI, education and income; Foster et al., 2018; Townsend et al., 2023; Figure 4). Full results demonstrating the associations between brain aging patterns and cognitive functions are presented in Appendix 1—table 7.

**Figure 5.**
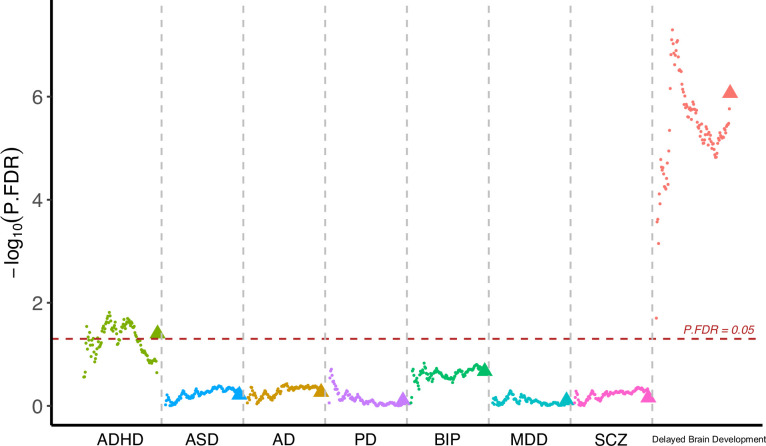
Participants with accelerated brain aging (brain aging pattern 2) had significantly increased genetic liability to ADHD and delayed brain development. Polygenic risk score (PRS) for ADHD, ASD, AD, PD, BIP, MDD, SCZ and delayed brain development (unpublished GWAS) were calculated at different p-value thresholds from 0.005 to 0.5 at an interval of 0.005. Vertical axis represents negative logarithm of P values comparing PRS in brain aging pattern 2 relative to pattern 1. Red horizontal dashed line indicates FDR corrected p value of 0.05. Colors represent traits and dots within the same color represent different p value thresholds. The trigonometric symbol indicates the average PRS across all p-value thresholds for the same trait. Figure 5—source data 1.Related to Figure 5.Participants with accelerated brain aging (brain aging pattern 2) had significantly increased genetic liability to ADHD and delayed brain development.

Having observed cognitive decline among participants with accelerated brain aging pattern, we next investigated whether brain aging patterns were associated with genetic vulnerability to major neuropsychiatric disorders. Since current GWAS are under-powered for attention-deficit/hyperactivity disorder (ADHD) and autism spectrum disorders (ASD) and the difficulty in identifying genetic variants was likely due to their polygenic nature, we calculated the corresponding polygenic risk scores (PRS) using multiple p value thresholds. This approach enabled robust investigation of the association between genetic susceptibility of neuropsychiatric disorders and brain imaging phenotypes. PRS for major neuro-developmental disorders including attention-deficit/hyperactivity disorder (ADHD) and autism spectrum disorders (ASD), neurodegenerative diseases including Alzheimer’s disease (AD) and Parkinson’s disease (PD), neuropsychiatric disorders including bipolar disorder (BIP), major depressive disorder (MDD), and schizophrenia (SCZ), and delayed structural brain development (GWAS from an unpublished longitudinal neuroimaging study) (Shi et al., 2023) were calculated for each participant using multiple p value thresholds (from 0.005 to 0.5 at intervals of 0.005) and results were then averaged over all thresholds (Figure 5). The primary GWAS datasets used for calculating the PRS were listed in Appendix 1—table 8. Overall, we observed increased genetic susceptibility to ADHD (p=0.040) and delayed brain development (p=1.48
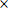
10^–6^) among participants with brain aging pattern 2 after FDR correction, while no statistically significant differences were observed for ASD, AD, PD, BIP, MDD, and SCZ (Figure 5). Details regarding the genetic liability to other common diseases and phenotypes using enhanced PRS from UK Biobank are displayed in Appendix 1tables 9 and 10.

### Genome Wide Association Studies (GWAS) identified significant genetic loci associated with accelerated brain aging

Having observed significant associations between brain aging patterns and cognitive performances / genetic liabilities to major neurodevelopmental disorders, we further investigated if there exist genetic variants contributing to individualized brain aging phenotype. We conducted genome-wide association studies (GWAS) using estimated total GMV at 60 years old as the phenotype. This phenotype was derived by adding individual specific deviations to the population averaged total GMV, thus providing additional information compared to studies using only cross-sectional neuroimaging phenotypes.

**Figure 6.**
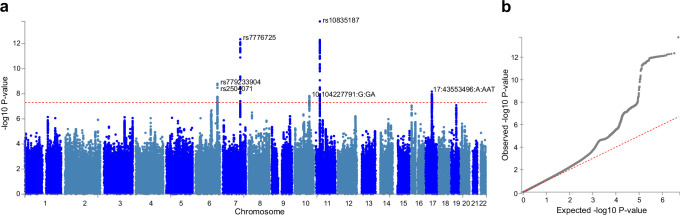
Genome-wide association study (GWAS) identified 6 independent SNPs associated with accelerated brain aging. Total GMV at 60 years old was estimated for each participant using mixed effect models allowing for individualized baseline GMV and GMV change rate, and was used as the phenotype in the GWAS. (**a**) At genome-wide significance level (p=5×10^–8^, red dashed line), rs10835187 and rs7776725 loci were identified to be associated with accelerated brain aging. (**b**) Quantile–quantile plot showed that the most significant p values deviate from the null, suggesting that results are not unduly inflated.

Six independent single nucleotide polymorphisms (SNPs) were identified at genome-wide significance level (p<5×10^–8^) (Figure 6) and were subsequently mapped to genes using NCBI, Ensembl and UCSC Genome Browser database (Appendix 1—table 11). Among them, two SNPs (rs10835187 and rs779233904) were also found to be associated with multiple brain imaging phenotypes in previous studies (Smith et al., 2021), such as regional and tissue volume, cortical area and white matter tract measurements. Compared to the GWAS using global gray matter volume as the phenotype, our GWAS revealed additional signal in chromosome 7 (rs7776725), which was mapped to the intron of FAM3C and encodes a secreted protein involved in pancreatic cancer (Grønborg et al., 2006) and Alzheimer’s disease (Liu et al., 2016). This signal was further validated to be associated with specific brain aging mode by another study using a data-driven decomposition approach (Smith et al., 2020). In addition, another significant loci (rs10835187, p=1.11×10^–13^) is an intergenic variant between gene LGR4-AS1 and LIN7C, and was reported to be associated with bone density and brain volume measurement (Smith et al., 2021; Estrada et al., 2012). *LIN7C* encodes the Lin-7C protein, which is involved in the localization and stabilization of ion channels in polarized cells, such as neurons and epithelial cells (Bohl et al., 2007; Kaech et al., 1998). Previous study has revealed the association of both allelic and haplotypic variations in the *LIN7C* gene with ADHD (Lanktree et al., 2008).

### Mirroring patterns between brain aging and brain development

**Figure 7.**
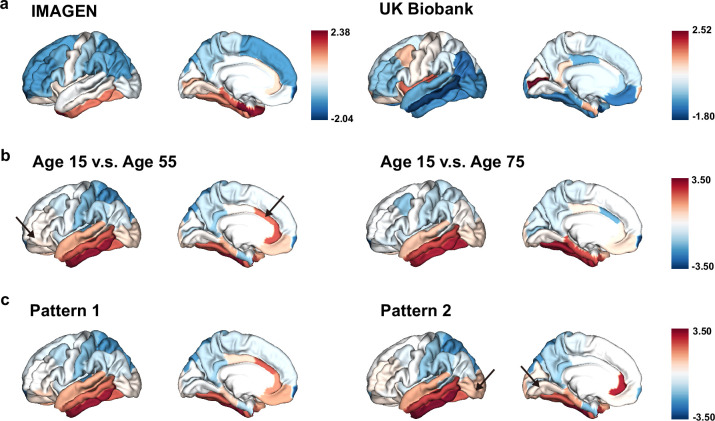
The ‘last in, first out’ mirroring patterns between brain development and brain aging. (**a**) The annual percentage volume change (APC) was calculated for each ROI and standardized across the whole brain in adolescents (IMAGEN, left) and mid-to-late aged adults (UK Biobank, right), respectively. For adolescents, ROIs of in red indicate delayed structural brain development, while for mid-to-late aged adults, ROIs in blue indicate accelerated structural brain aging. (**b**) Estimated APC in brain development versus early aging (55 years old, left), and versus late aging (75 years old, right). ROIs in red indicate faster GMV decrease during brain aging and slower GMV decrease during brain development, that is stronger mirroring effects between brain development and brain aging. (**c**) Mirroring patterns between brain development and brain aging were more manifested in participants with accelerated aging (brain aging pattern 2). The arrows point to ROIs with more pronounced mirroring patterns in each subfigure. Figure 7—source data 1.Related to Figure 7.The ‘last in, first out’ mirroring patterns between brain development and brain aging.

Having observed significant associations between brain aging and genetic susceptibility to neurodevelopmental disorders, we are now interested in examining the mirroring patterns between brain aging and brain development in the whole population, and whether these mirroring patterns were more pronounced in those with accelerated brain aging. Adolescents in the IMAGEN cohort showed more rapid GMV decrease in the frontal and parietal lobes, especially the frontal pole, superior frontal gyrus, rostral middle frontal gyrus, inferior parietal lobule and superior parietal lobule, while those in their mid-to-late adulthood showed more accelerated GMV decrease in the temporal lobe, including medial orbitofrontal cortex, inferior parietal lobule and lateral occipital sulcus (Figure 7a). The mirroring patterns (with slower GMV decrease during brain development and more rapid GMV decrease during brain aging) were particularly prominent in inferior temporal gyrus, caudal anterior cingulate cortex, fusiform cortex, middle temporal gyrus and rostral anterior cingulate cortex (Figure 7b). The regional mirroring patterns became weaker when we focus on late brain aging at age 75 years old, especially in the frontal lobe and cingulate cortex. Further, mirroring patterns were represented more prominently in participants with brain aging pattern 2, where stronger mirroring between brain aging and brain development was observed in frontotemporal area, including lateral occipital sulcus and lingual gyrus (Figure 7c).

### Gene expression profiles were associated with delayed brain development and accelerated brain aging

The Allen Human Brain Atlas (AHBA) transcriptomic dataset (http://human.brain-map.org) were used to obtain the spatial correlation between gene expression profiles across cortex and structural brain development/aging via partial least square (PLS) regression. The first PLS component explained 24.7% and 53.6% of the GMV change during brain development (estimated at age 15y, r_spearman_ = 0.51, P_permutation_ = 0.03) and brain aging (estimated at age 55y, r_spearman_ = 0.49, P_permutation_ = 1.5
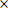
10^–4^), respectively. Seventeen of the 45 genes mapped to GWAS significant SNP were found in AHBA, with *LGR4* (r_spearman_ = 0.56, P_permutation_ <0.001) significantly associated with delayed brain development and *ESR1* (r_spearman_ = 0.53, P_permutation_ <0.001) and *FAM3C* (r_spearman_ = –0.37, P_permutation_ = 0.004) significantly associated with accelerated brain aging. *BDNF-AS* was positively associated with both delayed brain development and accelerated brain aging after spatial permutation test (Appendix 1—table 12 and 13).

**Figure 8.**
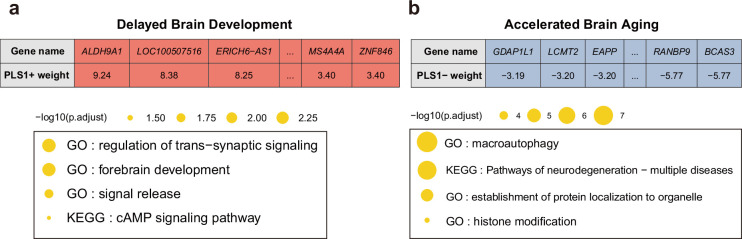
Functional enrichment of gene transcripts significantly associated with delayed brain development and accelerated brain aging. (**a**) 990 genes were spatially correlated with the first PLS component of delayed structural brain development, and were enriched for trans-synaptic signal regulation, forebrain development, signal release and cAMP signaling pathway. (**b**) 2293 genes were spatially correlated the first PLS component of accelerated structural brain aging, and were enriched for macroautophagy, pathways of neurodegeneration, establishment of protein localization to organelle and histone modification. Size of the circle represents number of genes in each term and P values were corrected using FDR for multiple comparisons. Figure 8—source data 1.Related to Figure 8.Functional enrichment of gene transcripts significantly associated with delayed brain development and accelerated brain aging. Each gene’s contribution to the first PLS component for IMAGEN and UKB. Figure 8—source data 2.Related to Figure 8.Functional enrichment of gene transcripts significantly associated with delayed brain development and accelerated brain aging. Gene set enrichment for genes spatially positively correlated with the first PLS component in IMAGEN and negatively correlated with the first PLS component in UKB.

Next, we screened the genes based on their contributions and effect directions to the first PLS components in brain development and brain aging. 990 and 2293 genes were identified to be positively associated with brain development and negatively associated with brain aging at FDR corrected p value of 0.005, respectively, representing gene expressions associated with delayed brain development and accelerated brain aging. These genes were then tested for enrichment of GO biological processes and KEGG pathways. Genes associated with delayed brain development showed significant enrichment in ‘regulation of trans-synaptic signaling’, ‘forebrain development’, ‘signal release’ and ‘cAMP signaling pathway’ (Figure 8a), and genes associated with accelerated brain aging showed significant enrichment in ‘macroautophagy’, ‘establishment of protein localization to organelle’, ‘histone modification’, and ‘pathways of neurodegeneration – multiple diseases’ (Figure 8b). Full results of the gene set enrichment analysis were provided in Appendix 1—figure 5. In summary, the analyses from using the databases of GO biological processes and KEGG Pathways indicate synaptic transmission as an important process in the common mechanisms of brain development and aging, and cellular processes (autophagy), as well as the progression of neurodegenerative diseases, are important processes in the mechanisms of brain aging.

## Discussion

In this study, we adopted a data-driven approach and revealed two distinct brain aging patterns using large-scale longitudinal neuroimaging data in mid-to-late adulthood. Compared to brain aging pattern 1, brain aging pattern 2 were characterized by a faster rate of GMV decrease, accelerated biological aging, cognitive decline, and genetic susceptibility to neurodevelopmental disorders. By integrating longitudinal neuroimaging data from adult and adolescent cohorts, we demonstrated the ‘last in, first out’ mirroring patterns between structural brain aging and brain development, and showed that the mirroring pattern was manifested in the temporal lobe and among participants with accelerated brain aging. Further, genome-wide association studies identified significant genetic loci contributing to accelerated brain aging, while spatial correlation between whole-brain transcriptomic profiles and structural brain aging / development revealed important gene sets associated with both accelerated brain aging and delayed brain development.

Brain aging is closely related to the onset and progression of neurodegenerative and neuropsychiatric disorders. Both neurodegenerative and neuropsychiatric disorders demonstrate strong inter-individual heterogeneity, which prevents the comprehensive understanding of their neuropathology and neurogenetic basis. Therefore, multidimensional investigation into disease subtyping and population clustering of structural brain aging are crucial in elucidating the sources of heterogeneity and neurophysiological basis related to the disease spectrum (Habes et al., 2020). In the last decades, major developments in the subtyping of Alzheimer’s disease, dementia and Parkinson’s disease, have provided new perspectives regarding their clinical diagnosis, treatment, disease progression and prognostics (Habes et al., 2020; Berg et al., 2021; Ferreira et al., 2020). While previous studies of brain aging mostly focused on the cross-sectional differences between cases and healthy controls, we here delineated the structural brain aging patterns among healthy participants using a novel data-driven approach that captured both cross-sectional and longitudinal trajectories of the whole-brain gray matter volume (Feczko et al., 2019; Poulakis et al., 2022). The two brain aging patterns identified using the above approach showed large differences in the rate of change in medial occipitotemporal gyrus, which is involved in vision, word processing, and scene recognition (Bogousslavsky et al., 1987; Epstein et al., 1999; Mechelli et al., 2000). Significant reduction of the gray matter volume and abnormal changes of the functional connectivity in this region were found in subjects with mild cognitive impairment (MCI) and AD, respectively (Chételat et al., 2005; Yao et al., 2010). Previous research on brainAGE (Elliott et al., 2021; Christman et al., 2020) (the difference between chronological age and the age predicted by the machine learning model of brain imaging data) showed that as a biomarker of accelerated brain aging, people with older brainAGE have accelerated biological aging and early signs of cognitive decline, which is consistent with our discoveries in this study. Our results support the establishment of a network connecting brain aging patterns with biological aging profiles involving multi-organ systems throughout the body (Tian et al., 2023). Since structural brain patterns might manifest and diverge decades before cognitive decline (Aljondi et al., 2019), subtyping of brain aging patterns could aid in the early prediction of cognitive decline and severe neurodegenerative and neuropsychiatric disorders.

Mirroring pattern between brain development and brain aging has long been hypothesized by postulating that phylogenetically newer and ontogenetically less precocious brain structures degenerate relatively early (Douaud et al., 2014). Early studies have reported a positive correlation between age-related differences of cortical volumes and precedence of myelination of intracortical fibers (Raz, 2000). Large differences in the patterns of change between adolescent late development and aging in the medial temporal cortex were previously found in studies of brain development and aging patterns (Tamnes et al., 2013). Here, we compared the annual volume change of the whole-brain gray matter during brain development and early / late stages of brain aging, and found that mirroring patterns are predominantly localized to the lateral / medial temporal cortex and the cingulate cortex. These cortical regions characterized by ‘last in, first out’ mirroring patterns showed increased vulnerability to the several neuropsychiatric disorders. For example, regional deficits in the superior temporal gyrus and medial temporal lobe were observed in schizophrenia (Honea et al., 2005), along with morphological abnormalities in the medial occipitotemporal gyrus (Schultz et al., 2010). Children diagnosed with ADHD had lower brain surface area in the frontal, cingulate, and temporal regions (Hoogman et al., 2019). Douaud et al., 2014 revealed a population transmodal network with lifespan trajectories characterized by the mirroring pattern of development and aging. We investigated the genetic susceptibility to individual-level mirroring patterns based on the lasting impact of neurodevelopmental genetic factors on brain (Fjell et al., 2015), demonstrating that those with more rapidly brain aging patterns have a higher risk of delayed development.

Identifying genes contributing to structural brain aging remains a critical step in understanding the molecular changes and biological mechanisms that govern age-related cognitive decline. Several genetic loci have been reported to be associated with brain aging modes and neurocognitive decline, many of which demonstrated global overlap with neuropsychiatric disorders and their related risk factors (Smith et al., 2020; Glahn et al., 2013; Brouwer et al., 2022). Here, we focused on the individual brain aging phenotype by estimating individual deviation from the population averaged total GMV and conducted genome-wide association analysis with this phenotype. Our approach identified six risk SNPs associated with accelerated brain aging, most of which could be further validated by previous studies using population averaged brain aging phenotypes. However, our approach revealed additional genetic signals and demonstrated genetic architecture underlying brain aging patterns overlap with bone density (Estrada et al., 2012; Zheng et al., 2015). In addition, molecular profiling of the aging brain has been thoroughly investigated among patients with neurogenerative diseases, but rarely conducted to shed light on the mirroring patterns among healthy participants. Analysis of the spatial correlation between gene expression profiles and structural brain development / aging further identified genes contributing to delayed brain development and accelerated brain aging. Specifically, expression of gene *BDNF-AS* was significantly associated with both processes. *BDNF-AS* is an antisense RNA gene and plays a role in the pathoetiology of non-neoplastic conditions mainly through the mediation of *BDNF* (Ghafouri-Fard et al., 2021). LGR4 (associated with delayed brain development) and FAM3C (associated with accelerated brain aging) identified in the spatial genetic association analysis also validated our findings in the GWAS.

There are several limitations in the current study that need to be addressed in future research. Firstly, the UK Biobank cohort, which we leveraged to identify population clustering of brain aging patterns, had a limited number of repeated structural MRI scans. Therefore, it remains challenging to obtain robust estimation of the longitudinal whole-brain GMV trajectory at the individual level. As a robustness check, we have calculated both intra-class correlation and variance of both random intercept and age slope to ensure appropriateness of the mixed effect models. Secondly, although aging is driven by numerous hallmarks, we have only investigated the association between brain aging patterns and biological aging in terms of telomere length and blood biochemical markers due to limitations of data access. Other dimensions of aging hallmarks and their relationship with structural brain aging need to be investigated in the future. Thirdly, our genomic analyses were restricted to ‘white British’ participants of European ancestry. The diversity of genomic analyses will continue to improve as the sample sizes of GWAS of non-European ancestry increase. Further, although the gene expression maps from Allen Human Brain Atlas enabled us to gain insights into the spatial coupling between gene expression profiles and mirroring patterns of the brain, the strong inter-individual variation of whole-brain gene expression levels and large temporal span of the human brain samples may lead to the inaccurate correspondence in the observed associations. Finally, we focused on structural MRIs in deriving brain aging patterns in this analysis, future investigations could consider other brain imaging modalities from a multi-dimensional perspective. Nevertheless, our study represents a novel attempt for population clustering of structural brain aging and validated the mirroring pattern hypothesis by leveraging large-scale adolescent and adult cohorts.

## Methods

### Participants

T1-weighted brain MRI images were obtained from 37,013 individuals aged 44–82 years old from UK Biobank (36,914 participants at baseline visit in 2014+, 4007 participants at the first follow-up visit in 2019+). All participants from UK Biobank provided written informed consent, and ethical approval was granted by the North West Multi-Center Ethics committee (https://www.ukbiobank.ac.uk/learn-more-about-uk-biobank/about-us/ethics) with research ethics committee (REC) approval number 16/NW/0274. Participants were excluded if they were diagnosed with severe psychiatric disorders or neurological diseases using ICD-10 primary and secondary diagnostic codes or from self-reported medical conditions at UK Biobank assessment center (see Appendix 1—tables 1 and 2). Data were obtained under application number 19542. A total of 1529 adolescents with structural MRI images were drawn from the longitudinal project IMAGEN (1463 at age 14, 1377 at age 19, and 1148 at age 23), of which the average number of MRI scans was 2.61 per adolescent. The lMAGEN study was approved by local ethics research committees of King’s College London, University of Nottingham. Trinity College Dublin, University of Heidelberg, Technische Universität Dresden, Commissariat à l'Énergie Atomique et aux Énergies Alternatives, and University Medical Center at the University of Hamburg in compliance with the Declaration of Helsinki (Association, 2013). Informed consent was given by all participants and a parent/guardian of each participant.

### MRI acquisition

Quality-controlled T1-weighted neuroimaging data from UK Biobank and IMAGEN were processed using FreeSurfer v6.0. Detailed imaging processing pipeline can be found online for UK Biobank (https://biobank.ctsu.ox.ac.uk/crystal/crystal/docs/brain_mri.pdf) and IMAGEN (https://github.com/imagen2/imagen_mri; Schumann et al., 2010; Imagen, 2020). Briefly, cortical gray matter volume (GMV) from 33 regions in each hemisphere were generated using Desikan–Killiany Atlas (Desikan et al., 2006), and total gray matter volume (TGMV), intracranial volume (ICV) and subcortical volume were derived from ASEG atlas (Fischl et al., 2002 See Appendix 1—table 3). Regional volume was averaged across left and right hemispheres. To avoid deficient segmentation or parcellation, participants with TGMV, ICV or regional GMV beyond 4 standard deviations from the sample mean were considered as outliers and removed from the following analyses.

### Identification of longitudinal brain aging patterns

Whole-brain GMV trajectory was estimated for each participant in 40 brain regions of interest (ROIs) (33 cortical regions and 7 subcortical regions), using mixed effect regression model with fixed linear and quadratic age effects, random intercept and random age slope. Covariates include sex, assessment center, handedness, ethnic, and ICV. Models with random intercept and with both random intercept and random age slope were compared using AIC, BIC and evaluation of intra-class correlation (ICC). Results suggested that random age slope model should be chosen for almost all ROIs (Appendix 1—table 14). Deviation of regional GMV from the population average was calculated for each participant at age 60 years and dimensionality reduction was conducted via principal component analysis (PCA). The first 15 principal components explaining approximately 70% of the total variations of regional GMV deviation were used in multivariate k-means clustering. Optimal number of clusters was chosen using both elbow diagram and contour coefficient (Appendix 1—figure 6). Rates of volumetric change for total gray matter and each ROI were estimated using generalized additive mixed effect models (GAMM) with fixed cubic splines of age, random intercept and random age slope, which incorporates both cross-sectional between-subject variation and longitudinal within-subject variation from 40,921 observations and 37,013 participants. Covariates include sex, assessment center, handedness, ethnic, and ICV. We also applied PCA and locally linear embedding (LLE; Roweis and Saul, 2000) to the adjusted GMV ROIs in order to map the high-dimensional imaging-derived phenotypes to a low-dimensional space for stratification visualisation. The GMV of 40 ROIs at baseline were linearly adjusted for sex, assessment center, handedness, ethnic, ICV, and second-degree polynomial in age to be consistent with the whole-brain GMV trajectory model.

### Association between brain aging patterns and biological aging, cognitive decline and genetic susceptibilities of neuropsychiatric disorders

Individuals with Z-standardized leucocyte telomere length (Codd et al., 2021) and blood biochemistry (which were used to calculate PhenoAge (Levine et al., 2018) that characterizes biological aging) outside 4 standard deviations from the sample mean were excluded for better quality control. A total of 11 cognitive tests performed on the touchscreen questionnaire were included in the analysis. More information about the cognitive tests is provided in Supplementary Information. Comparisons of biological aging (leucocyte telomere length, PhenoAge) and cognitive function were conducted among participants with different brain aging patterns using both unadjusted and adjusted multivariate regression models with Bonferroni / FDR correction. Polygenic Risk Scores (PRS) were calculated for autism spectrum disorder (ASD), attention deficit hyperactivity disorder (ADHD), Alzheimer’s disease (AD), Parkinson’s Disease (PD), bipolar disorder (BIP), major depressive disorder (MDD), schizophrenia (SCZ), and delayed brain development using GWAS summary statistics (Shi et al., 2023) at multiple p value thresholds (from 0.005 to 0.5 at intervals of 0.005, and 1), with higher p value thresholds incorporating larger number of independent SNPs. After quality control of genotype and imaging data, PRSs were generated for 25,861 participants on UK Biobank genotyping data. SNPs were pruned and clumped with a cutoff r^2^ ≥ 0.1 within a 250 kb window. All calculations were conducted using PRSice v2.3.5 (Choi and O’Reilly, 2019). Enhanced PRS from UK Biobank Genomics for multiple diseases were also tested. Detailed instructions for calculating enhanced PRS in UK Biobank can be found in research of Thompson et al., 2022 Comparisons of neuropsychiatric disorders were conducted among participants with different brain aging patterns using t test with FDR correction. All statistical tests were two-sided.

### Genome wide association Study to identify SNPs associated with brain aging patterns

We performed Genome-wide association studies (GWAS) on individual deviations of total GMV relative to the population average at 60 years using PLINK 2.0 (Chang et al., 2015). Variants with missing call rates exceeding 5%, minor allele frequency below 0.5% and imputation INFO score less than 0.8 were filtered out after the genotyping quality control for UK Biobank Imputation V3 dataset. Among the 337,138 unrelated ‘white British’ participants of European ancestry included in our study, 25,861 with recent UK ancestry and accepted genotyping and imaging quality control were included in the GWAS. The analyses were further adjusted for age, age2, sex, assessment center, handedness, ethnic, ICV, and the first 10 genetic principal components. Genome-wide significant SNPs (p<5
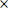
10^–8^) obtained from the GWAS were clumped by linkage disequilibrium (LD) (r^2^ <0.1 within a 250 kb window) using UKB release2b White British as the reference panel. We subsequently performed gene-based annotation in FUMA (Watanabe et al., 2017) using genome-wide significant SNPs and SNPs in close LD (r^2^ ≥ 0.1) using Annotate Variation (ANNOVAR) on Ensemble v102 genes (Wang et al., 2010).

### Mirroring patterns between brain aging and brain development

To validate the ‘last in, first out’ mirroring hypothesis, we evaluated the structural association between brain development and brain aging. Longitudinal neuroimaging data from 1529 adolescents in the IMGAEN cohort and 3908 mid-to-late adulthood in the UK Biobank cohort were analyzed. Annual percentage volume change (APC) for each ROI was calculated among individuals with at least two structural MRI scans by subtracting the baseline GMV from follow-up GMV and dividing by the number of years between baseline and follow-up visits. Region-specific APC was regressed on age using smoothing spline with cross validated degree of freedom. Estimated APC for each ROI was obtained at age 15y for adolescents and at age 55y (early aging) and 75y (late aging) for participants in UK Biobank. Region-specific APC during adolescence (or mid-to-late adulthood) was then standardized across all cortical regions to create the brain development (or aging) map. Finally, the brain development map and brain aging map were compared to assess the mirroring pattern for each ROI in the overall population and across different aging subgroups.

### Gene expression analysis

The Allen Human Brain Atlas (AHBA) dataset (http://human.brain-map.org), which comprises gene expression measurements in six postmortem adults (age 24–57y) across 83 parcellated brain regions (Hawrylycz et al., 2012; Markello et al., 2021), were used to identify gene expressions significantly associated with structural brain development and aging. The expression profiles of 15,633 genes were averaged across donors to form a 83
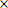
15,633 transcriptional matrix and partial least squares (PLS) regression was adopted for analyzing the association between regional change rate of gray matter volume and gene expression profiles. Specifically, estimated regional APC at 15 (obtained from IMAGEN cohort) and 55 years old (obtained from UK Biobank) were regressed on the high-dimensional gene expression profiles upon regularization. Associations between the first PLS component and estimated APC during brain development and brain aging were tested by spatial permutation analysis (10,000 times; Váša et al., 2018). Additionally, gene expression profiles of genes mapped to GWAS significant SNP were extracted from AHBA. The association between gene expression profiles of mapped genes and estimated APC during brain development and aging was also tested by spatial permutation analysis. Statistical significance of each gene’s contribution to the first PLS component was tested with standard error calculated using bootstrap (Li et al., 2021; Morgan et al., 2019; Romero-Garcia et al., 2020), and genes significantly associated with delayed brain development and accelerated brain aging were selected. Enrichment of Kyoto Encyclopedia of Genes and Genomes (KEGG) pathways and gene ontology (GO) of biological processes for these selected genes were analyzed using R package clusterProfiler (Yu et al., 2012). All statistical significances were corrected for multiple testing using FDR.

### Code availability

R version 4.2.0 was used to perform statistical analyses. FreeSurfer version 6.0 was used to process neuroimaging data. lme4 1.1 in R version 4.2.0 was used to perform longitudinal data analyses. PRSice version 2.3.5 (https://choishingwan.github.io/PRSice/; Choi and O’Reilly, 2019) was used to calculate the PRS. PLINK 2.0 (https://www.cog-genomics.org/plink/2.0/) and FUMA version 1.5.6 (https://fuma.ctglab.nl/) were used to perform genome-wide association analysis, and ANNOVAR was used to perform gene-based annotation. AHBA microarray expression data were processed using abagen toolbox version 0.1.3 (https://doi.org/10.5281/zenodo.5129257). The rotate_parcellation code used to perform a spatial permutation test of a parcellated cortical map: https://github.com/frantisekvasa/rotate_parcellation (Váša, 2023; Váša et al., 2018). Code for PLS analysis and bootstrapping to estimate PLS weights are available at https://github.com/KirstieJane/NSPN_WhitakerVertes_PNAS2016/tree/master/SCRIPTS (Whitaker, 2016; Whitaker et al., 2016). clusterProfiler 4.6 in R version 4.2.0 was used to analyze gene-set enrichment.

## Figures and Tables

**Appendix 1—figure 1.**
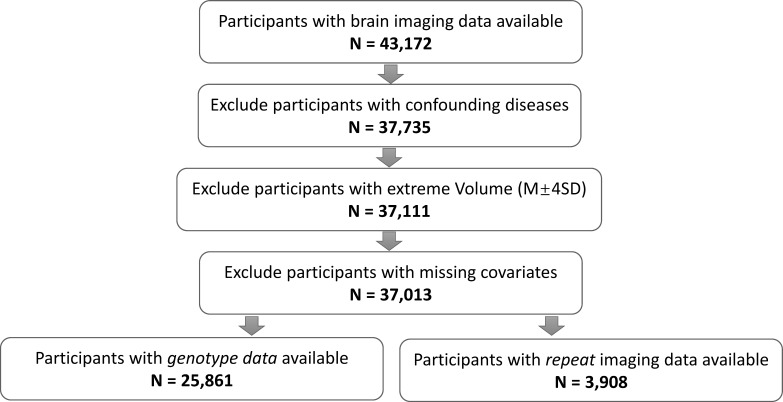
The sample selection workflow.

**Appendix 1—figure 2.**
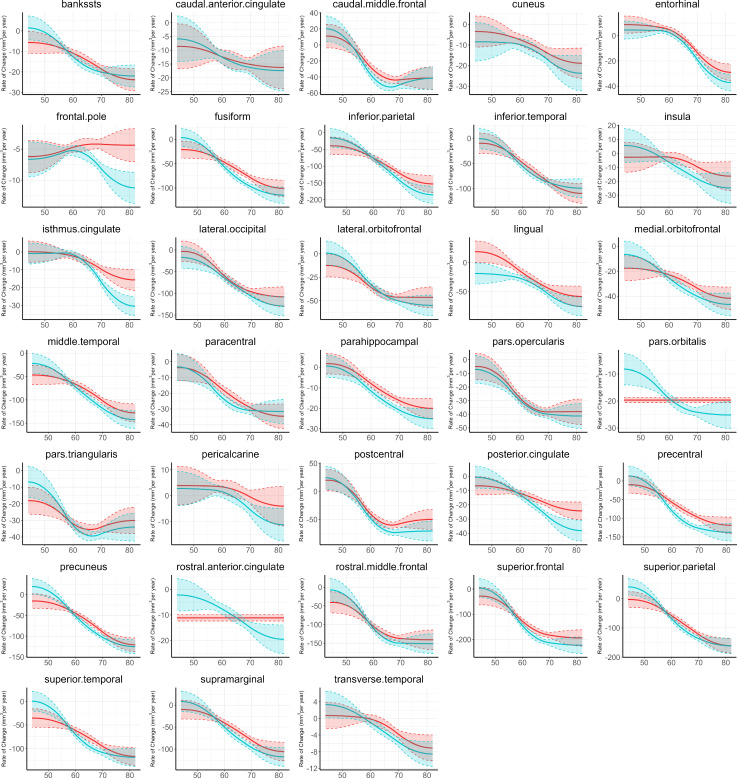
Estimated rates of change in regional volumes for 33 bilateral brain regions.

**Appendix 1—figure 3.**
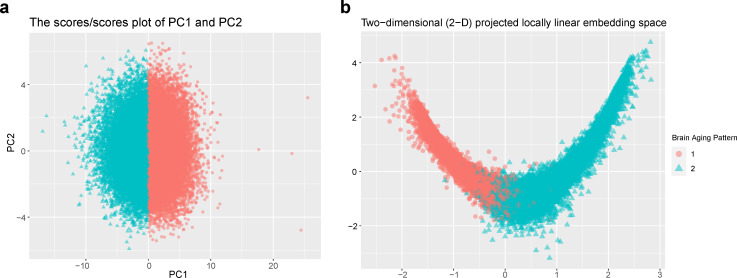
Stratification of the identified brain aging patterns using linear and non-linear dimensionality reduction methods.

**Appendix 1—figure 4.**
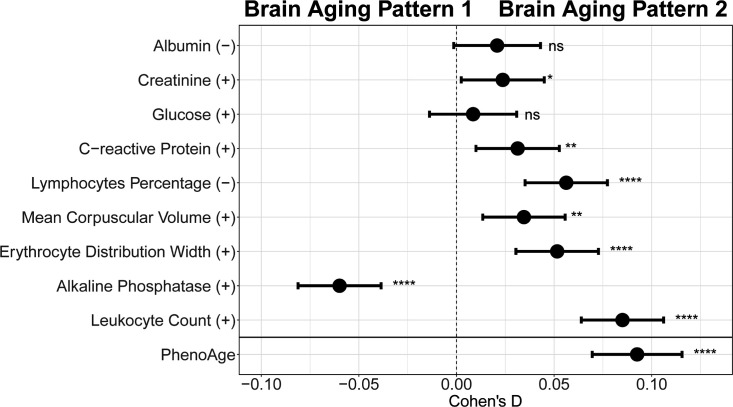
Effect size for comparing each individual blood biochemical metric (used to calculate the PhenoAge) between participants with brain aging patterns 1 and 2.

**Appendix 1—figure 5.**
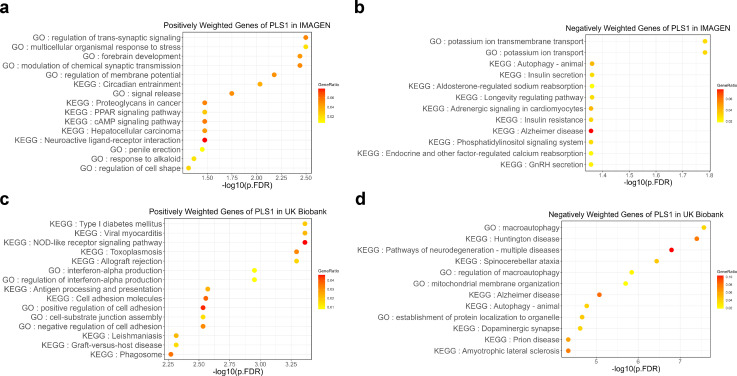
Gene set enrichment of Kyoto Encyclopedia of Genes and Genomes (KEGG) pathways and gene ontology (GO) of biological processes.

**Appendix 1—figure 6.**
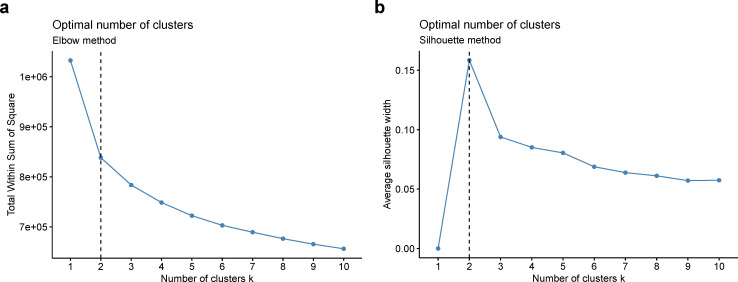
Optimal number of clusters was chosen using elbow method (**a**) and silhouette method (**b**).

## Data Availability

The summary statistics of GWAS for individual deviations of total GMV is available at https://doi.org/10.5061/dryad.jh9w0vtmn. All the UK Biobank data used in the study are available at https://www.ukbiobank.ac.uk. The IMAGEN project data are available at https://imagen-project.org (Schumann et al., 2010). GWAS summary statistics used to calculate the PRS are available in the Appendix 1—table 8. Human gene expression data are available in the Allen Human Brain Atlas dataset: https://human.brainmap.org (Hawrylycz et al., 2012). Source data files have been provided for Figures 2—5, 7 and 8, which contain the numerical data used to generate the figures. Summary statistics of the GWAS for delayed brain development by Shi et al are available at https://delayedneurodevelopment.page.link/amTC. The following dataset was generated: DuanH ShiR KangJ 2024The summary statistics of GWAS for individual deviations of total GMVDryad10.5061/dryad.jh9w0vtmn The following previously published datasets were used: NallsMA BlauwendraatC VallergaCL 2019Parkinson’s disease or first degree relation to individual with Parkinson’s diseaseGWAS CatalogGCST009325 SullivanP 2021adhd2019figshare10.6084/m9.figshare.14671965 SullivanP 2019asd2019figshare10.6084/m9.figshare.14671989 JansenIE SavageJE WatanabeK 2019Summary statistics for Genome-wide meta-analysis identifies new loci and functional pathways influencing Alzheimer's disease riskVrije Universiteit Research Drivel7aiRr1UEgdoJfZ10.1038/s41588-018-0311-9PMC683667530617256 SullivanP 2023bip2021_noUKBBfigshare10.6084/m9.figshare.22564402 AdamsMJ 2023MDD2 (MDD2018) GWAS sumstats w/o UKBBfigshare10.6084/m9.figshare.21655784 SullivanP 2022scz2022figshare10.6084/m9.figshare.19426775

## Additional files

MDAR checklist

## Appendix 1

Population clustering of structural brain aging and its association with brain development.

### Supplementary method

#### Cognitive assessment

##### Reaction time

This cognitive function test is based on 12 rounds of the card-game 'Snap'. The participant is shown two cards at a time; if both cards are the same, they press a button-box that is on the table in front of them as quickly as possible. The score used for analysis is mean time to correctly identify matches (UK Biobank data field 20023), which is the mean duration to first press of snap-button summed over rounds in which both cards matched.

##### Numeric memory

The participant was shown a 2-digit number to remember. The number then disappeared and after a short while they were asked to enter the number onto the screen. The number became one digit longer each time they remembered correctly (up to a maximum of 12 digits). This test is available for a subset of participants. The score used for analysis is maximum digits remembered correctly (UK Biobank data field 4282), which is longest number correctly recalled during the numeric memory test.

##### Fluid intelligence / reasoning

'Fluid intelligence' is defined as the capacity to solve problems that require logic and reasoning ability, independent of acquired knowledge. The participant has 2 min to complete as many questions as possible from the test. This test was incorporated into the touchscreen towards the end of recruitment. The score used for analysis is fluid intelligence score (UK Biobank data field 20016), which is a simple unweighted sum of the number of correct answers given to the 13 fluid intelligence questions. Participants who did not answer all of the questions within the allotted 2 min limit are scored as zero for each of the unattempted questions.

##### Trail making

The participant was presented with sets of digits/letters in circles scattered around the screen and asked to click on them sequentially according to a specific algorithm. The scores used for analysis are duration to complete numeric path (trail #1) (UK Biobank data field 6348) and duration to complete alphanumeric path (trail #2) (UK Biobank data field 6350).

##### Matrix pattern completion

The participant was presented with a series of matrix pattern blocks with an element missing and asked to select the element that best completed the pattern from a range of displayed choices. The score used for analysis is number of puzzles correctly solved (UK Biobank data field 6373).

##### Symbol digit substitution

The participant was presented with one grid linking symbols to single-digit integers and a second grid containing only the symbols. They were then asked to indicate the numbers attached to each of the symbols in the second grid using the first one as a key. The score used for analysis is number of symbol digit matches made correctly (UK Biobank data field 23324).

#### Tower rearranging

The participant was presented with an illustration of three pegs (towers) on which three differently-colored hoops had been placed. The were then asked to indicate how many moves it would take to re-arrange the hoops into another specific position. The score used for analysis is number of puzzles correct (UK Biobank data field 21004).

##### Paired associate learning

In the paired associate learning test the participants were shown 12 pairs of words (for 30 s in total) then, after an interval (in which they did a different test), presented with the first word of 10 of these pairs and asked to select the matching second word from a choice of four alternatives. The words were presented in the order: huge, happy, tattered, old, long, red, sulking, pretty, tiny and new. The score used for analysis is number of word pairs correctly associated (UK Biobank data field 21097), which is the number of word pairs correctly associated out of 10 attempts.

##### Prospective memory

Early in the touchscreen cognitive section, the participant is shown the message "At the end of the games we will show you four colored shapes and ask you to touch the Blue Square. However, to test your memory, we want you to actually touch the Orange Circle instead." The score used for analysis is prospective memory result (UK Biobank data field 20018), which condenses the results of the prospective memory test into 3 groups (“0”: instruction not recalled, either skipped or incorrect, “1”: correct recall on first attempt, “2”: correct recall on second attempt). We divided the test results into two groups for simplicity of analysis: “0” indicating no correct recall, and the combination of “1” and “2” indicating correct recall.

##### Pairs matching

Participants are asked to memorize the position of as many matching pairs of cards as possible. The cards are then turned face down on the screen and the participant is asked to touch as many pairs as possible in the fewest tries. Multiple rounds were conducted. The first round used 3 pairs of cards and the second 6 pairs of cards. In the pilot phase an additional (i.e. third) round was conducted using six pairs of cards. However this was dropped from the main study as the extra set of results were very similar to the second and not felt to add significant new information. The score used for analysis is number of incorrect matches in round 2 (UK Biobank data field 399.2).

**Appendix 1—table 1.** ICD-10 primary and secondary diagnostic codes for exclusion criteria.

Condition	Code
Malignant neoplasm	C70, C71
Dementia	F00, F01, F02, F03, F04
Mental and behavioural disorders due to psychoactive substance use	F10-F19
Schizophrenia, schizotypal and delusional disorders	F20-F29
Mood [affective] disorders	F30-F39
Mental retardation	F70-F79
Disorders of psychological development	F80-F89
Hyperkinetic disorders	F90
Inflammatory diseases of the central nervous system	G00-G09
Systemic atrophies primarily affecting the central nervous system	G10, G11, G122, G13
Extrapyramidal and movement disorders	G20, G21, G22, G23
Other degenerative diseases of the nervous system	G30-G32
Demyelinating diseases of the central nervous system	G35-G37
Episodic and paroxysmal disorders	G40, G41, G45, G46
Infantile cerebral palsy	G80
Cerebrovascular diseases	I60-I69
Down’s syndrome	Q90
Intracranial injury	S06

**Appendix 1—table 2.** Self-reported illness codes for exclusion criteria.

Field ID	Condition	Code
20001	Brain cancer/primary malignant brain tumour	1032
	Meningeal cancer/malignant meningioma	1031
20002	Benign neuroma	1683
	Brain abscess/intracranial abscess	1245
	Brain haemorrhage	1491
	Cerebral aneurysm	1425
	Cerebral palsy	1433
	Chronic/degenerative neurological problem	1258
	dementia/alzheimers/cognitive impairment	1263
	Encephalitis	1246
	Epilepsy	1264
	Fracture skull/head	1626
	Head injury	1266
	Ischaemic stroke	1583
	Meningioma/benign meningeal tumour	1659
	Meningitis	1247
	Motor Neurone Disease	1259
	Multiple Sclerosis	1261
	Nervous system infection	1244
	Neurological injury/trauma	1240
	Other demyelinating disease (not Multiple Sclerosis)	1397
	Other neurological problem	1434
	Parkinson’s Disease	1262
	Spina Bifida	1524
	Stroke	1081
	Subarachnoid haemorrhage	1086
	Subdural haemorrhage/haematoma	1083
	Transient ischaemic attack	1082

**Appendix 1—table 3.** Cortical and subcortical brain regions.

Desikan–Killiany Atlas
bankssts caudal anterior cingulate caudal middle frontal cuneus entorhinal fusiform inferior parietal inferior temporal isthmus cingulate lateral occipital lateral orbitofrontal lingual medial orbitofrontal middle temporal parahippocampal paracentral pars opercularis pars orbitalis pars triangularis pericalcarine postcentral posterior cingulate precentral precuneus rostral anterior cingulate rostral middle frontal superior frontal superior parietal superior temporal supramarginal frontal pole transverse temporal insula
**ASEG Atlas**
thalamus proper caudate putamen pallidum hippocampus amygdala accumbens area

**Appendix 1—table 4.** Loadings matrix for the first 15 principal components.

	PC1	PC2	PC3	PC4	PC5	PC6	PC7	PC8	PC9	PC10	PC11	PC12	PC13	PC14	PC15
**Cortical**															
bankssts	0.12	–0.25	0.01	0.30	–0.13	0.01	0.09	0.34	0.04	0.05	–0.10	0.16	–0.09	–0.20	0.02
caudal.anterior.cingulate	0.12	–0.06	–0.02	0.13	0.39	0.28	0.13	–0.09	–0.37	0.24	0.05	0.03	–0.04	–0.13	0.07
caudal.middle.frontal	0.15	0.01	–0.10	–0.11	0.34	–0.21	0.10	0.06	0.03	–0.28	–0.23	–0.20	0.24	0.00	0.25
cuneus	0.13	0.47	–0.01	0.06	–0.01	0.00	0.07	0.12	0.01	0.07	0.12	–0.05	–0.09	0.08	–0.07
entorhinal	0.09	0.07	0.13	0.08	0.10	0.17	–0.53	–0.18	0.06	0.04	–0.19	–0.10	0.13	0.01	–0.04
fusiform	0.18	–0.03	0.01	0.13	–0.02	0.02	–0.20	–0.02	0.15	0.30	–0.01	–0.28	–0.23	0.08	0.21
inferior.parietal	0.15	–0.18	0.01	0.39	–0.09	0.01	0.14	–0.04	0.16	0.05	–0.20	–0.02	–0.15	–0.02	0.02
inferior.temporal	0.16	–0.13	0.01	0.26	–0.01	0.15	–0.04	–0.10	0.14	–0.08	–0.08	–0.25	0.20	0.29	–0.01
isthmus.cingulate	0.15	0.23	0.02	0.11	–0.14	–0.03	0.08	–0.03	–0.23	–0.07	–0.22	0.16	0.43	0.04	0.11
lateral.occipital	0.16	0.30	0.00	0.12	–0.02	0.02	0.05	0.10	0.16	0.18	0.06	–0.23	–0.23	0.12	0.17
lateral.orbitofrontal	0.22	–0.04	–0.08	–0.17	–0.05	0.15	–0.02	–0.15	0.09	–0.02	–0.01	0.40	–0.01	0.22	0.17
lingual	0.12	0.43	0.03	0.14	–0.01	–0.02	0.07	0.11	–0.01	–0.03	–0.10	0.16	0.02	–0.09	–0.08
medial.orbitofrontal	0.21	–0.06	–0.06	–0.06	–0.05	0.10	–0.01	–0.13	0.18	–0.06	0.13	0.17	0.05	0.01	–0.25
middle.temporal	0.17	–0.17	–0.01	0.27	–0.14	0.10	0.21	0.25	0.09	–0.15	–0.09	0.04	0.07	0.05	–0.11
parahippocampal	0.12	0.09	0.13	0.05	0.06	0.14	–0.46	0.02	–0.02	0.09	–0.22	0.09	0.03	–0.32	0.02
paracentral	0.19	–0.05	–0.10	–0.07	0.18	–0.23	–0.11	–0.06	0.01	0.03	–0.02	0.06	–0.35	–0.01	–0.35
pars.opercularis	0.16	–0.02	–0.06	–0.23	–0.19	0.10	0.14	0.01	–0.16	–0.13	–0.43	–0.29	–0.16	–0.06	0.00
pars.orbitalis	0.18	0.02	–0.05	–0.17	–0.11	0.24	0.03	–0.17	0.23	0.08	0.01	0.41	–0.14	0.05	0.24
pars.triangularis	0.16	0.02	–0.03	–0.35	–0.26	0.23	0.13	–0.05	–0.06	–0.06	–0.25	–0.17	–0.21	–0.08	–0.05
pericalcarine	0.10	0.47	–0.01	0.08	–0.01	0.01	0.09	0.13	–0.02	–0.01	0.02	0.08	–0.03	0.03	–0.17
postcentral	0.20	–0.04	–0.11	0.06	0.16	–0.29	–0.09	0.12	0.08	0.03	–0.09	0.10	0.04	0.01	0.04
posterior.cingulate	0.18	–0.08	–0.04	0.02	0.24	0.09	0.08	–0.02	–0.30	0.01	–0.07	0.10	–0.05	–0.12	–0.31
precentral	0.21	–0.01	–0.10	–0.08	0.28	–0.29	–0.09	0.08	0.15	–0.05	–0.15	0.13	–0.06	0.05	0.12
precuneus	0.20	0.02	–0.02	0.13	–0.24	–0.26	0.01	–0.35	–0.20	–0.04	0.06	–0.04	0.00	0.03	0.07
rostral.anterior.cingulate	0.15	–0.07	–0.07	0.05	0.31	0.21	0.15	–0.05	–0.30	0.13	0.11	–0.06	0.00	0.18	0.15
rostral.middle.frontal	0.20	0.02	–0.05	–0.05	0.10	0.14	0.22	–0.11	0.17	–0.11	0.24	–0.14	0.19	–0.22	0.14
superior.frontal	0.22	–0.03	–0.09	–0.21	0.14	–0.13	–0.01	0.06	0.20	–0.18	0.06	–0.11	0.02	0.00	–0.16
superior.parietal	0.17	0.02	–0.05	0.14	–0.18	–0.29	0.01	–0.45	–0.16	0.00	0.14	–0.08	–0.08	–0.06	0.04
superior.temporal	0.21	–0.13	–0.02	–0.06	–0.10	0.01	–0.07	0.40	–0.07	0.13	0.24	–0.05	0.02	–0.06	0.08
supramarginal	0.17	–0.15	–0.08	0.06	–0.24	–0.23	–0.13	0.03	–0.21	0.04	0.11	0.10	0.14	–0.08	–0.06
frontal.pole	0.16	0.03	–0.10	0.01	0.00	0.16	0.03	–0.13	0.28	0.05	0.28	–0.16	0.26	–0.38	–0.22
transverse.temporal	0.15	–0.01	–0.10	–0.27	–0.17	–0.10	–0.16	0.25	–0.22	0.19	0.24	–0.10	0.09	0.04	0.16
insula	0.19	–0.08	0.01	–0.22	–0.12	0.10	–0.07	0.11	–0.06	0.15	–0.08	0.00	0.19	0.16	–0.23
**Subcortical**															
thalamus.proper	0.09	–0.02	0.31	–0.08	0.03	–0.08	0.08	0.00	0.02	–0.21	0.11	0.10	–0.19	–0.37	0.33
caudate	0.06	–0.03	0.32	–0.12	0.02	–0.10	0.15	–0.02	0.11	0.31	–0.11	0.09	0.27	0.14	0.12
putamen	0.08	–0.03	0.42	–0.12	0.03	–0.13	0.13	–0.03	0.05	0.22	–0.05	–0.14	0.07	–0.02	–0.17
pallidum	0.03	–0.03	0.42	–0.07	0.00	–0.13	0.16	–0.06	0.04	0.20	–0.07	0.00	0.04	–0.20	–0.06
hippocampus	0.12	0.01	0.33	0.04	–0.02	0.11	–0.20	0.08	–0.16	–0.39	0.09	0.03	–0.11	–0.03	0.05
amygdala	0.13	–0.03	0.31	0.05	–0.01	0.12	–0.13	0.09	–0.07	–0.35	0.25	–0.09	–0.02	0.24	–0.01
accumbens.area	0.11	–0.05	0.31	–0.02	0.12	–0.07	0.11	–0.04	0.00	–0.03	0.10	0.09	–0.14	0.34	–0.18

**Appendix 1—table 5.** Baseline and demographic characteristics for participants in the total population and stratified by brain aging patterns.

	Total (n=37,013)	Pattern 1 (n=18,929)Pattern 2 (n=18,084)	
**Age (years), mean (SD**)	63.9 (7.63)	63.9 (7.64)	63.8 (7.63)
**Female, n (%**)	19,958 (53.9)	10,117 (53.4)	9,841 (54.4)
**Ethnicity, n (%**)			
**White**	34,219 (92.5)	17,509 (92.5)	16,710 (92.4)
**Mixed**	1,137 (3.1)	573 (3.0)	564 (3.1)
**Asian or Asian British**	1,210 (3.3)	612 (3.2)	598 (3.3)
**Other**	447 (1.2)	235 (1.2)	212 (1.2)
**Smoking status, n (%)** *			
**Never smoker**	23,633 (64.4)	12,269 (65.4)	11,364 (63.4)
**Previous smoker**	12,213 (33.3)	6,085 (32.4)	6,128 (34.2)
**Current smoker**	833 (2.3)	414 (2.2)	419 (2.3)
**TDI, mean (SD)** ^†^	–1.94 (2.69)	–1.97 (2.66)	–1.90 (2.71)
**BMI (kg/m^2^), mean (SD)** ^‡^	26.4 (4.32)	26.3 (4.17)	26.5 (4.46)
**Years of Schooling, mean (SD)** ^§^	16.8 (4.32)	16.9 (4.29)	16.8 (4,35)

TDI = Townsend Deprivation Index, BMI = Body Mass Index.

* Missing 334.

† Missing 36.

‡ Missing 1937.

§ Missing 337.

**Appendix 1—table 6.** Associations between results of biological aging biomarkers and subgroups stratified by whole-brain TGMV trajectories. Cohen’s d measures the standardized difference of means between brain aging pattern 2 and brain aging pattern 1.

Biological aging biomarkers	Brain aging pattern 1		Brain aging pattern 2			
	N	Mean(SD)	N	Mean(SD)		
LTL	17,691	0.083 (0.98)	16,876	0.055 (0.97)		
PhenoAge	15,228	41.35 (8.17)	14,323	41.58 (8.32)		
Biological aging biomarkers	Unadjusted			Adjusted		
	Cohen’s d (95% CI)	p	P.Bonferroni	Cohen’s d (95% CI)	p	P.Bonferroni
LTL	–0.028 (-0.049,–0.007)	0.009	0	–0.030 (-0.051,–0.009)	0.006	0.011
PhenoAge	0.027 (0.004, 0.050)	0.019	0	0.092 (0.070, 0.116)	3.05E-15	6.11E-15

**Appendix 1—table 7.** Associations between results of cognitive function tests and subgroups stratified by whole-brain TGMV trajectories. Cohen’s d measures the standardized difference of means between brain aging pattern 2 and brain aging pattern 1.

Cognitive functions	Brain aging pattern 1		Brain aging pattern 2			
	N	Mean(SD)	N	Mean(SD)		
Reaction time	17,749	–594.70 (108.15)	16,831	–594.68 (110.50)		
Numeric memory	13,350	6.82 (1.26)	12,346	6.72 (1.27)		
Fluid intelligence	17,580	6.73 (2.06)	16,612	6.53 (2.04)		
Trail making A	13,052	–224.50 (84.82)	12,036	–227.56 (84.85)		
Trail making B	12,743	–563.06 (246.74)	11,753	–576.55 (260.75)		
Matrix pattern completion	13,064	8.07 (2.11)	12,064	7.91 (2.14)		
Symbol digit substitution	13,077	19.08 (5.15)	12,056	18.87 (5.35)		
Tower rearranging	12,952	9.96 (3.20)	11,958	9.83 (3.23)		
Paired associate learning	13,184	7.01 (2.59)	12,198	6.88 (2.65)		
Prospective memory	17,831	N/A	16,949	N/A		
Pairs matching	17,840	–3.58 (2.90)	16,956	–3.67 (2.94)		
Cognitive functions	Unadjusted			Adjusted		
	Cohen’s d (95% CI)	P	P.FDR	Cohen’s d (95% CI)	P	P.FDR
Reaction time	0.000 (-0.021, 0.021)	0.99	0.99	0.006 (-0.016, 0.028)	0.61	0.61
Numeric memory	–0.082 (-0.106,–0.057)	5.97E-11	3.28E-10	–0.080 (-0.106,–0.055)	8.99E-10	4.95E-09
Fluid intelligence	–0.102 (-0.123,–0.080)	5.94E-21	6.54E-20	–0.99 (-0.121,–0.077)	3.30E-18	3.63E-17
Trail making A	–0.036 (-0.061,–0.011)	0.004	0.006	–0.050 (-0.074,–0.024)	1.46E-04	2.29E-04
Trail making B	–0.053 (-0.078,–0.028)	3.16E-05	8.68E-05	–0.067 (-0.093,–0.041)	6.16E-07	1.69E-06
Matrix pattern completion	–0.076 (-0.101,–0.051)	1.84E-09	6.74E-09	–0.078 (-0.104,–0.052)	3.67E-09	1.35E-08
Symbol digit substitution	–0.040 (-0.065,–0.015)	0.002	0.002	–0.053 (-0.079,–0.027)	6.15E-05	1.13E-04
Tower rearranging	–0.041 (-0.066,–0.016)	0.001	0.002	–0.049 (-0.075,–0.023)	2.18E-04	3.00E-04
Paired associate learning	–0.051 (-0.076,–0.027)	4.64E-05	1.02E-04	–0.054 (-0.079,–0.028)	4.89E-05	1.08E-04
Prospective memory	OR: 0.943 (0.891, 0.999)	0.047	0.052	OR: 0.940 (0.883, 1.000)	0.052	0.057
Pairs matching	–0.029 (-0.050,–0.008)	0.006	0.008	–0.033 (-0.055,–0.011)	0.003	0.004

**Appendix 1—table 8.** Genome-wide association study details. Loci associated with risk were thresholded at p<5×10^–8^, then distance-based clumping was used to define independently significant loci.

Study	Number cases	Number controls	Number of genome-wide independently significant loci	Download link
Attention deficit hyperactivity disorder Demontis et al., 2019	20,183	35,191	12	https://figshare.com/ndownloader/files/28169253
Autism spectrum disorder Grove et al., 2019	18,381	27,969	5	https://figshare.com/ndownloader/files/28169292
Alzheimer’s disease Jansen et al., 2019	71,880	383,378	25	https://ctg.cncr.nl/software/summary_statistics
Parkinson’s disease Nalls et al., 2019	37,688, and 18,618 (proxy-cases)	1,417,791	90	https://drive.google.com/file/d/1FZ9UL99LAqyWnyNBxxlx6qOUlfAnublN/view?usp=sharing
Bipolar disorder Mullins et al., 2021	41,917	371,549	64	https://figshare.com/ndownloader/files/40036705
Major depressive disorder Wray et al., 2018	135,458	344,901	44	https://figshare.com/ndownloader/files/39504667
Schizophrenia Trubetskoy et al., 2022	76,755	243,649	287	https://figshare.com/ndownloader/files/34517828
Delayed Brain Development Shi et al., 2023	7662 (proxy phenotype, continuous)		1	https://delayedneurodevelopment.page.link/amTC

**Appendix 1—table 9.** Polygenic Risk Scores comparisons between two subgroups. Data supporting these scores were obtained either entirely from external GWAS data (the Standard PRS set). The bold P values reflect significance after FDR correction.

Trait	n1	n2	statistic	p	p.adjust
AAM	18,429	17,586	–2.218	0.027	0.080
AMD	18,429	17,586	1.753	0.080	0.169
AD	18,429	17,586	0.735	0.462	0.616
AST	18,429	17,586	–0.861	0.389	0.543
AF	18,429	17,586	–0.100	0.920	0.945
BD	18,429	17,586	3.557	3.75E-04	0.002
BMI	18,429	17,586	–3.309	0.001	0.005
CRC	18,429	17,586	–0.544	0.586	0.703
BC	18,429	17,586	–3.140	0.002	0.008
CVD	18,429	17,586	–2.104	0.035	0.091
CED	18,429	17,586	1.046	0.296	0.484
CAD	18,429	17,586	–1.588	0.112	0.202
CD	18,429	17,586	–0.094	0.925	0.945
EOC	18,429	17,586	–2.183	0.029	0.080
EBMDT	18,429	17,586	–11.343	<1.00E-20	<1.00E-20
HBA1C_DF	18,429	17,586	–2.948	0.003	0.013
HEIGHT	18,429	17,586	6.658	2.81E-11	3.37E-10
HDL	18,429	17,586	0.884	0.377	0.543
HT	18,429	17,586	–3.539	4.02E-04	0.002
IOP	18,429	17,586	–1.605	0.109	0.202
ISS	18,429	17,586	–2.383	0.017	0.056
LDL_SF	18,429	17,586	–0.686	0.492	0.627
MEL	18,429	17,586	2.025	0.043	0.103
MS	18,429	17,586	–0.069	0.945	0.945
OP	18,429	17,586	12.029	<1.00E-20	<1.00E-20
PD	18,429	17,586	1.456	0.145	0.249
POAG	18,429	17,586	–0.856	0.392	0.543
PC	18,429	17,586	–0.240	0.810	0.941
PSO	18,429	17,586	–1.781	0.075	0.169
RA	18,429	17,586	–2.437	0.015	0.053
SCZ	18,429	17,586	0.158	0.874	0.945
SLE	18,429	17,586	1.695	0.090	0.180
T1D	18,429	17,586	0.666	0.505	0.627
T2D	18,429	17,586	–5.523	3.35E-08	3.02E-07
UC	18,429	17,586	0.883	0.377	0.543
VTE	18,429	17,586	–0.170	0.865	0.945

**Appendix 1—table 10.** Polygenic Risk Scores comparisons between two subgroups. Data supporting these scores were obtained external and internal UK Biobank data (the Enhanced PRS set). The bold p values reflect significance after FDR correction.

Trait	n1	n2	statistic	p	p.adjust
AAM	3,407	3,409	–1.708	0.088	0.344
AMD	3,407	3,409	0.547	0.584	0.931
AD	3,407	3,409	0.756	0.450	0.820
APOEA	3,407	3,409	0.023	0.982	0.993
APOEB	3,407	3,409	0.119	0.905	0.968
AST	3,407	3,409	0.112	0.911	0.968
AF	3,407	3,409	1.306	0.192	0.600
BD	3,407	3,409	0.561	0.575	0.931
BMI	3,407	3,409	–0.976	0.329	0.730
CRC	3,407	3,409	0.984	0.325	0.730
BC	3,407	3,409	–0.995	0.320	0.730
CAL	3,407	3,409	–1.786	0.074	0.326
CVD	3,407	3,409	–0.009	0.993	0.993
CED	3,407	3,409	1.280	0.200	0.600
CAD	3,407	3,409	0.231	0.818	0.961
DOA	3,407	3,409	–0.326	0.745	0.961
EOC	3,407	3,409	–2.167	0.030	0.155
EBMDT	3,407	3,409	–6.111	1.04E-09	2.65E-08
EGCR	3,407	3,409	0.413	0.680	0.961
EGCY	3,407	3,409	–0.210	0.834	0.961
HBA1C_DF	3,407	3,409	0.130	0.896	0.968
HEIGHT	3,407	3,409	4.351	1.38E-05	2.35E-04
HDL	3,407	3,409	0.294	0.769	0.961
HT	3,407	3,409	–0.884	0.377	0.743
IOP	3,407	3,409	–2.366	0.018	0.151
ISS	3,407	3,409	0.066	0.947	0.986
LDL_SF	3,407	3,409	–0.193	0.847	0.961
MEL	3,407	3,409	2.659	0.008	0.080
MS	3,407	3,409	–2.293	0.022	0.151
OTFA	3,407	3,409	0.318	0.750	0.961
OSFA	3,407	3,409	0.770	0.441	0.820
OP	3,407	3,409	6.484	9.54E-11	4.87E-09
PD	3,407	3,409	1.041	0.298	0.730
PDCL	3,407	3,409	0.663	0.507	0.862
PHG	3,407	3,409	0.392	0.695	0.961
PFA	3,407	3,409	0.495	0.621	0.932
POAG	3,407	3,409	–1.083	0.279	0.730
PC	3,407	3,409	0.675	0.500	0.862
PSO	3,407	3,409	–2.234	0.026	0.151
RMNC	3,407	3,409	0.501	0.617	0.932
RHR	3,407	3,409	–2.865	0.004	0.053
RA	3,407	3,409	0.297	0.766	0.961
SCZ	3,407	3,409	–0.880	0.379	0.743
SGM	3,407	3,409	–0.224	0.823	0.961
SLE	3,407	3,409	1.458	0.145	0.493
TCH	3,407	3,409	0.191	0.848	0.961
TFA	3,407	3,409	0.892	0.372	0.743
TTG	3,407	3,409	1.212	0.226	0.640
T1D	3,407	3,409	1.771	0.077	0.326
T2D	3,407	3,409	–2.218	0.027	0.151
VTE	3,407	3,409	–1.613	0.107	0.390

**Appendix 1—table 11.** Most significant single-variant associations (p < 510^-8^) detected in the GWAS analyses. Six independent SNPs at genome-wide significance level were identified by linkage disequilibrium (LD) clumping (r2 < 0.1 within a 250 kb window). The location (chromosome [chr] and base position [bp]), alleles (A1 = effect allele and A2 = other allele), effect (β) and its standard error (β SE) with respect to A1, and association p-values from regression model of the variants are given, along with functional consequences of SNPs on gene by performing ANNOVAR.

SNP	A1	A2	p-value	β	β SE	Location (chr:bp)	Gene symbol	Position relative to gene
rs10835187	C	T	1.70e-14	–0.02558	0.003333	11:27505677	LGR4, LIN7C	intergenic
rs7776725	C	T	4.47e-13	–0.02640	0.003644	7:121033121	FAM3C	intronic
rs779233904	AAC	A	1.57e-09	0.02083	0.003449	6:151910404	CCDC170	intronic
rs2504071	T	C	3.34e-09	0.01959	0.003311	6:152084862	ESR1	intronic
17:43553496:A:AAT	A	AAT	6.65e-09	–0.02472	0.004261	17:43553496	PLEKHM1	intronic
10:104227791:G:GA	GA	G	1.48e-08	–0.01889	0.003334	10:104227791	TMEM180	intronic

**Appendix 1—table 12.** Association between gene expression profiles of mapped genes and estimated APC during brain development. The bold p values reflect significance after the spatial permutation test.

Gene Symbol	Spearman's ρ	p value	P.permutation
ACTR1A	0.096	0.440	0.328
ARHGAP27	0.051	0.684	0.445
ARL17B	0.047	0.705	0.452
BDNF-AS	0.256	0.038	0.038
CCDC170	0.268	0.030	0.069
ESR1	0.021	0.870	0.483
FAM3C	–0.096	0.444	0.356
KANSL1	–0.262	0.034	0.073
KANSL1-AS1	0.067	0.594	0.313
LGR4	0.558	1.78E-06	2.50E-04
LIN7C	0.036	0.775	0.464
LRRC37A4P	–0.272	0.027	0.148
MAPT	0.024	0.846	0.405
PLEKHM1	–0.276	0.025	0.109
SPPL2C	0.116	0.351	0.189
STH	–0.147	0.238	0.277
SUFU	0.407	0.001	0.028

**Appendix 1—table 13.** Association between gene expression profiles of mapped genes and estimated APC during brain aging. The bold p values reflect significance after the spatial permutation test.

Gene Symbol	Spearman's ρ	Pvalue	P.permutation
ACTR1A	–0.235	0.058	0.052
ARHGAP27	0.486	4.48E-05	5.50E-04
ARL17B	0.090	0.473	0.240
BDNF-AS	0.490	3.68E-05	1.50E-04
CCDC170	0.206	0.098	0.075
ESR1	0.532	6.02E-06	1.50E-04
FAM3C	–0.366	0.003	0.005
KANSL1	0.213	0.086	0.078
KANSL1-AS1	–0.262	0.034	0.059
LGR4	0.070	0.576	0.348
LIN7C	0.177	0.154	0.120
LRRC37A4P	0.143	0.250	0.165
MAPT	–0.287	0.020	0.022
PLEKHM1	0.202	0.104	0.080
SPPL2C	0.211	0.089	0.059
STH	–0.001	0.997	0.490
SUFU	–0.036	0.773	0.373

**Appendix 1—table 14.** Model evaluation results using relative measures: AIC, BIC, likelihood ratio test and intra-class correlation (ICC).

model	AIC	BIC	lrtest	ICC_adjusted	ICC_unadjusted
lmer_intr_thalamus.proper	79444.24	79591.29	6.45E-15	0.8875	0.3958
lmer_slope_thalamus.proper	79382.89	79547.24	6.45E-15	0.8889	0.3986
lmer_intr_caudate	95845.48	95992.54	2.68E-71	0.9543	0.6824
lmer_slope_caudate	95524.49	95688.84	2.68E-71	0.9564	0.6817
lmer_intr_putamen	92106.06	92253.12	2.40E-42	0.9398	0.5946
lmer_slope_putamen	91918.4	92082.75	2.40E-42	0.9424	0.5933
lmer_intr_pallidum	90500.3	90647.36	4.21E-42	0.8859	0.5116
lmer_slope_pallidum	90313.76	90478.12	4.21E-42	0.8862	0.5093
lmer_intr_hippocampus	89833.92	89980.97	1.37E-08	0.9309	0.5505
lmer_slope_hippocampus	89801.71	89966.06	1.37E-08	0.9329	0.5505
lmer_intr_amygdala	89976.98	90124.03	4.01E-06	0.8697	0.4898
lmer_slope_amygdala	89956.13	90120.48	4.01E-06	0.8713	0.4897
lmer_intr_accumbens.area	96936.19	97083.24	1.69E-14	0.8228	0.5295
lmer_slope_accumbens.area	96876.77	97041.12	1.69E-14	0.8233	0.5310
lmer_intr_bankssts	97981.96	98129.01	0.001818	0.9339	0.6798
lmer_slope_bankssts	97973.34	98137.69	0.001818	0.9354	0.6814
lmer_intr_caudal.anterior.cingulate	109133.2	109280.3	0.131507	0.8638	0.7653
lmer_slope_caudal.anterior.cingulate	109133.2	109297.5	0.131507	0.8644	0.7659
lmer_intr_caudal.middle.frontal	93118.13	93265.19	8.31E-06	0.9227	0.5929
lmer_slope_caudal.middle.frontal	93098.74	93263.09	8.31E-06	0.9246	0.5949
lmer_intr_cuneus	101801.4	101948.5	0.014429	0.9242	0.7256
lmer_slope_cuneus	101796.9	101961.3	0.014429	0.9245	0.7262
lmer_intr_entorhinal	109404.9	109551.9	1.63E-14	0.8013	0.6908
lmer_slope_entorhinal	109345.4	109509.7	1.63E-14	0.8037	0.6928
lmer_intr_fusiform	87545.88	87692.93	9.17E-05	0.9139	0.5062
lmer_slope_fusiform	87531.28	87695.63	9.17E-05	0.9163	0.5074
lmer_intr_inferior.parietal	89044.43	89191.48	5.89E-14	0.9374	0.5564
lmer_slope_inferior.parietal	88987.51	89151.86	5.89E-14	0.9419	0.5603
lmer_intr_inferior.temporal	84066.47	84213.52	5.73E-07	0.9384	0.4956
lmer_slope_inferior.temporal	84041.73	84206.08	5.73E-07	0.9405	0.4977
lmer_intr_isthmus.cingulate	92442.12	92589.17	0.127191	0.9275	0.5862
lmer_slope_isthmus.cingulate	92442	92606.35	0.127191	0.9284	0.5869
lmer_intr_lateral.occipital	89550.4	89697.45	0.003943	0.9121	0.5273
lmer_slope_lateral.occipital	89543.33	89707.68	0.003943	0.9129	0.5282
lmer_intr_lateral.orbitofrontal	86224.13	86371.18	1.29E-08	0.8466	0.4323
lmer_slope_lateral.orbitofrontal	86191.79	86356.14	1.29E-08	0.8497	0.4345
lmer_intr_lingual	102605.2	102752.2	0.041242	0.9181	0.7268
lmer_slope_lingual	102602.8	102767.2	0.041242	0.9181	0.7272
lmer_intr_medial.orbitofrontal	89954.36	90101.41	2.25E-06	0.7832	0.4219
lmer_slope_medial.orbitofrontal	89932.35	90096.7	2.25E-06	0.7872	0.4241
lmer_intr_middle.temporal	83331.01	83478.06	0.0019	0.9177	0.4615
lmer_slope_middle.temporal	83322.48	83486.83	0.0019	0.9195	0.4629
lmer_intr_parahippocampal	108997.1	109144.2	4.99E-05	0.8686	0.7639
lmer_slope_parahippocampal	108981.3	109145.6	4.99E-05	0.8690	0.7639
lmer_intr_paracentral	98058.63	98205.68	0.015686	0.8695	0.5958
lmer_slope_paracentral	98054.32	98218.67	0.015686	0.8705	0.5960
lmer_intr_pars.opercularis	96829.05	96976.1	1.20E-12	0.9354	0.6658
lmer_slope_pars.opercularis	96778.15	96942.5	1.20E-12	0.9396	0.6698
lmer_intr_pars.orbitalis	96989.61	97136.67	1.39E-05	0.8785	0.5892
lmer_slope_pars.orbitalis	96971.25	97135.6	1.39E-05	0.8805	0.5912
lmer_intr_pars.triangularis	96637.58	96784.63	4.55E-18	0.9402	0.6710
lmer_slope_pars.triangularis	96561.72	96726.07	4.55E-18	0.9439	0.6748
lmer_intr_pericalcarine	105115.9	105263	0.022841	0.9429	0.8190
lmer_slope_pericalcarine	105112.4	105276.7	0.022841	0.9441	0.8200
lmer_intr_postcentral	91605.6	91752.65	0.000549	0.8764	0.5189
lmer_slope_postcentral	91594.59	91758.94	0.000549	0.8789	0.5203
lmer_intr_posterior.cingulate	96853.73	97000.78	2.21E-19	0.8913	0.6014
lmer_slope_posterior.cingulate	96771.82	96936.17	2.21E-19	0.8949	0.6022
lmer_intr_precentral	91186.59	91333.64	2.61E-12	0.8478	0.4864
lmer_slope_precentral	91137.25	91301.6	2.61E-12	0.8504	0.4864
lmer_intr_precuneus	84734.58	84881.63	1.23E-08	0.9088	0.4708
lmer_slope_precuneus	84702.16	84866.51	1.23E-08	0.9126	0.4730
lmer_intr_rostral.anterior.cingulate	95688.68	95835.73	2.64E-12	0.9093	0.6097
lmer_slope_rostral.anterior.cingulate	95639.36	95803.72	2.64E-12	0.9122	0.6129
lmer_intr_rostral.middle.frontal	80873.84	81020.89	2.57E-17	0.9137	0.4354
lmer_slope_rostral.middle.frontal	80801.44	80965.79	2.57E-17	0.9191	0.4395
lmer_intr_superior.frontal	79730.6	79877.65	1.25E-11	0.8921	0.4038
lmer_slope_superior.frontal	79684.38	79848.73	1.25E-11	0.8972	0.4060
lmer_intr_superior.parietal	92895.95	93043	8.55E-07	0.8899	0.5487
lmer_slope_superior.parietal	92872.01	93036.36	8.55E-07	0.8934	0.5512
lmer_intr_superior.temporal	86495.24	86642.29	0.003021	0.9148	0.4959
lmer_slope_superior.temporal	86487.64	86651.99	0.003021	0.9168	0.4971
lmer_intr_supramarginal	87390.39	87537.44	3.67E-13	0.9263	0.5221
lmer_slope_supramarginal	87337.12	87501.47	3.67E-13	0.9320	0.5258
lmer_intr_frontal.pole	110426.3	110573.4	1.95E-65	0.6907	0.5868
lmer_slope_frontal.pole	110132.3	110296.7	1.95E-65	0.6957	0.5882
lmer_intr_transverse.temporal	102753.2	102900.2	0.032413	0.9130	0.7247
lmer_slope_transverse.temporal	102750.3	102914.7	0.032413	0.9144	0.7258
lmer_intr_insula	88693.2	88840.26	7.61E-14	0.8263	0.4416
lmer_slope_insula	88636.79	88801.14	7.61E-14	0.8290	0.4420
